# Commentary: Evidence for Replacement of an Infected Synthetic by a Biological Mesh in Abdominal Wall Hernia Repair

**DOI:** 10.3389/fsurg.2017.00059

**Published:** 2017-10-11

**Authors:** Ekaterini Christina Tampaki, Athanasios Tampakis, Konstantinos Kontzoglou, Gregory Kouraklis

**Affiliations:** ^1^Second Department of Propaedeutic Surgery, Laiko General Hospital, School of Medicine, National and Kapodistrian University of Athens, Athens, Greece; ^2^Department of Visceral Surgery, Basel University Hospital, Basel, Switzerland

**Keywords:** hernia, mesh infection, biological mesh, mesh replacement, mesh complication

It was a pleasure to go through the manuscript by Montgomery et al. because it provides stimulating arguments in favor of using biologic meshes and replacing infected synthetic ones in difficult abdominal wall reconstruction (AWR), whereas it brings up excellent discussion topics on the subject (1).

First, we certainly agree that biologic meshes are being used with increased frequency in many fields and, indeed, the outcomes are perceived to be better than those for traditional polymer-based prosthetic mesh replacement materials. However, we believe that the use of biological grafts increased rapidly without clear clinical evidence of efficacy and, therefore, we would like to highlight that selection of the proper implant is always crucial along with careful consideration of patient characteristics related to prosthetic will as this could effectively lead us to decreased complication rates, readmissions, and number of postoperative visits.

Interestingly, the same side of the coin as suggested above is being presented in recent clinical reports focusing on the successful use of light weighted, macroporous synthetic meshes in contaminated ventral hernia reconstructions, showing that in contaminations with *Staphylococcus aureus* and *Escherichia coli*, the biologic meshes proved to be less resistant compared to reduced-weight synthetics and, therefore, raising the question whether biologics should be questioned in contaminated ventral hernia reconstruction (2).

Furthermore, a highly anticipated multicenter prospective double-blinded randomized controlled trial by Rosen et al. examining material safety, efficacy, and cost effectiveness wants to demonstrate that the use of a macroporous light-weight polypropylene mesh is much more cost effective in comparison to the use of a biologic mesh (3). As suggested above, with currently >200 meshes being commercially available in the United States, it is significant to highlight strength and weaknesses of materials used and always explore possibilities of combining them so as to take advantage of their benefits. As far as synthetic meshes are concerned, tensile strength, porosity, elasticity, and fabrication method are significant. Excessive tensile strength leads often to inflammation, material contraction, and further postoperative pain, whereas the various pore sizes influence the meshes incorporation into the surrounding tissues. Additionally, knitting materials are more porous and flexible, while weaving materials appear stronger. Permanent meshes have demonstrated higher infection and fistula rates, increased recurrence rates, and even cases of small bowel obstruction, while expanded meshes primarily used in vascular grafts and in abdominal surgeries present higher hernia recurrence rates and shrinkage in size. A combination of the above create “composite” meshes, approved for clinical use, while lastly absorbable meshes degrade fully and can be used in contaminated fields (4).

On the other hand, the collagen matrix of biological grafts acts regeneratively promoting new collagen deposition, impacting a better biocompatibility and immunogenic ability and leading to decreased infection resistance. As a consequence, excessive scaring and graft encapsulation can be avoided. Moreover, the chemically cross-linked collagen matrix can resist degradation for several years from various enzymes like collagenases. Although in case studies where infection occurred, a graft removal was seldom necessary (5), follow-up studies showed a high incidence of laxity, eventration, and recurrent herniation with the authors highlighting again the insufficiency of high-quality evidence regarding biological mesh use in ventral hernia repair (5).

Indisputably, both biologic and synthetic materials present with advantages and disadvantages. In an attempt to overcome them, both have been lately combined into the release of a hybrid mesh appearing very promising as it is expected the biologic component to protect the synthetic one from infections leading to a biologic component replacement and the final synthetic mesh incorporation into the tissue host, with a diminished risk of fistulization (5).

The latest COBRA study showed a significant advantage of biosynthetic absorbable meshes related to long-term recurrence and quality of life in patients with more complex situations of ventral hernia repair, presenting their use as a good alternative over biologic and permanent synthetic mesh use (6). The above also highlights that besides material use, other factors related to mesh complications play a vital role including the various surgical techniques used, which influence long-term results, several patient and technical factors. More specifically, while it is known that permanent synthetic meshes are related to higher infection rates, therefore, contraindicating their use in contaminated fields, a recent meta-analysis showed that an overall infection rate reaching up to 5% combined with certain patient risk factors such as smoking, American Society of Anesthesiologists score >3, and emergency operation, worsened the chances of infection (7). Moreover, the impact of demographics such as patients body mass index as well as certain risk factors such as hernia grade, hernia size, and past bariatric surgery have been shown as predictive factors of recurrence (8). The above emphasizes the absolute necessity of proper implant selection along with careful consideration of patient characteristics related to prosthetic will.

In the latest retrospective review by Chamieh et al., the authors concluded that synthetic meshes are not inferior to biologic meshes when working on similar cohort patients in contaminated fields. More specifically, patients length of stay was 4 days longer concerning biological meshes, whereas re-admission rates were 52.9% in the biologic group versus a 45.8% in the synthetic group. Surgical site infections and recurrence with re-admitions were less frequent for biological meshes (38.9 versus 55.6%, respectively). The overall infection rate was more frequent in the biologic group showing, however, a less frequent microbiology of Gram-positive bacteria (50 and 29.2% for synthetic versus 39 and 63%, respectively) (9).

Concluding, based on a late systematic review regarding costs and efficacy of biologic mesh implants in AWR, their expense cannot be fully justified, whereas the evidence remains insufficient to determine a favorable correlation between cost and clinical benefits of the biological materials (10). Therefore, we believe that until high-level of evidence coming from randomized clinical trials demonstrates superiority of biological materials, the expense associated with their use cannot be confirmed and, therefore, it is highly risky to suggest the superiority and selection of these materials given their cost, and their preference over synthetic meshes in difficult AWR.

## Author Contributions

ECT conceived of the idea and drafted the manuscript. AT helped draft the manuscript. KK and GK helped to revise the manuscript. All authors read and approved the final manuscript.

## Conflict of Interest Statement

The authors declare that the research was conducted in the absence of any commercial or financial relationships that could be construed as a potential conflict of interest. The reviewer KB and handling editor declared their shared affiliation.

## Abbreviations

AWR, abdominal wall reconstruction; BMI, body mass index; SSIs, surgical site infections.
